# *The Organon* and Materia Medica Club of the Bay Cities of California

**Published:** 1896-06

**Authors:** W. E. Ledyard

**Affiliations:** Secretary


### *THE ORGANON* AND MATERIA MEDICA CLUB OF THE BAY CITIES OF CALIFORNIA.

The regular semi-monthly meeting was held at the office of Dr. George H. Martin, 606 Sutter Street, San Francisco, Friday evening, February 21st, 1896.

Members present: Drs. J. M. Selfridge, G. J. Augur, M. T. Wilson, M. F. Underwood, and A. McNeil.

The meeting was called to order at 8.15 o'clock by the President, Dr. J. M. Selfridge. The minutes of the previous meeting were read and approved.

Dr. Selfridge then read from *The Organon,* Sections 63 and 64.

### Discussion.

Dr. McNeil—Much has been said about primary and secondary effects of drugs. Hempel says if the disease symptoms are like the primary action of the drug, we are to give a high potency. Hale says the opposite to this. Dunham covers both of these opinions, and says he has seen one remedy cure diarrhœa and constipation in one day. I think Dunham is right. I never make any difference in the potency to suit the primary or secondary action of drugs.

Dr. Selfridge—My recollection of Hempel is that he believes that the drug antidotes the morbific cause of the disease. He says if you wish to use high potencies, commence with Arsenic. Sulphur is the remedy for early morning diarrhœa, and may be used for constipation as well.

Dr. Wilson—Hahnemann claims that the secondary effect of a drug is the curative effect.

Dr. McNeil—The secondary effect of a drug may meet either diarrhœa or constipation, and it makes no difference whether high or low potencies are used.

Section 65 was read.

DISCUSSION.

Dr. Wilson—This is a corroboration of what he said in the previous section.

Dr. McNeil—Is it not strange that the allopaths pay attention to both actions of drugs?

Dr. Selfridge—They are doing it more now.

Dr. Augur—Take for instance Ipecac. They give it to produce vomiting, and to cure it.

Dr. McNeil—Is is only occasionally that they make use of both actions.

Section 66 was read.

DISCUSSION.

Dr. McNeil—The only meaning to the word "alterative" used in this sense would be *active*. It is not used in the same sense as the old-school men use the word.

Dr. Augur—It would certainly not be used as the old-school men use the word "alterative."

Dr. McNeil—Do they not apply it to the remedies we recognize as antipsorics, as Iodide of Potassium, etc.?

Dr. Augur—Yes.

Dr. Selfridge—I understand that an alterative in their way brings the system under its effects by giving small doses.

Dr. Augur—The action seems to be scarcely recognizable to them. It is a slow, mild action.

Section 67 was read.

DISCUSSION.

Dr. Augur—In the practice of medicine I believe in applying the Golden Rule as often as possible. I think that there are very few of us who, if suffering acute pain, and could be relieved by an injection of Morphine, would not use it in preference to waiting for the action of the homœopathic remedy. In such a case in our practice I think we should do as we would like to have done by us, and prescribe for such a condition as we would prescribe for ourselves.

Dr. McNeil—The illustration is not a fair one. When a man is suffering from acute pain he is not a fit subject to give medicine to himself, because his judgment is more or less affected. Therefore, to use the Golden Rule, as Dr. Augur says, and by doing so to use palliatives when our patients are suffering pain, would not be the proper thing to do at all.

Dr. Augur—Dr. McNeil has taken a little different view of it. The patient is not competent to prescribe for himself, but in the case I have in mind, I think the doctor would agree with the patient. Take, for instance, nephritic colic. There are remedies which ameliorate that condition, but I do not know of anything which will relieve the condition as quickly as a hypodermic of Morphine, and if I had it and Morphine would relieve my condition, I would take it rather than wait for the action of the homœopathic remedy. If after trying the homœopathic remedy you do not get relief, is it not better to use a hypodermic of Morphine to blunt the sensibilities for the time being?

Dr. Selfridge—I think the homœopathic remedy will do this if you really get the right remedy.

Dr. Augur—I have only practiced Homœopathy three years, but I am sure that there is no one who has more confidence and faith in the efficacy of homœopathie remedies than I have. We have never had a proving producing nephritic colic, and in such a case I would be afraid to wait for the action of a remedy.

Dr. Selfridge—In my own case at one time, I thought *Rhus* the remedy and took it. The pain stopped, but came back again. I lost my grip and had a small injection of Morphine.

Dr. McNeil—I am very glad this question was brought up. Dr. Augur made a mistake when he said we have never had a proving producing nephritic colic. We never designed to produce diseases. The assumption is that the passage of a calculus is necessarily painful. It is not necessarily painful of itself, but when this morbid condition is present it produces spasm of the ureter which causes the pain. We all know that when the agency is most excruciating, an ordinary dose of an opiate will not relieve the patient. I gave three-fourths of a grain at at one time before it would blunt the pain.

Dr. Augur—One-half grain relieves in my case.

Dr. McNeil—One-half grain is nothing. At any rate, after the calculus has passed into the bladder the pain is over, and you have given the patient one-fourth to three-quarters of a grain of Morphine. To give this to a well man would be a very serious thing. When the pain is gone the patient is almost a well man, but the results of your treatment might be very serious. In one case of gall-stone colic, I gave several drugs without any effect. I could not get a clear picture of the case. All at once the patient said: "How strange it is that every time the pains come on my hands tingle." I gave one dose of Secale-cornutum$^{200}$ and in half an hour the patient was free from pain. The passage of calculus was still going on and yet the pain stopped.

Dr. Augur—When I practiced allopathy it was my custom never to give more than one-fourth grain of Morphine at one time, but repeated the dose. In such a case as Dr. McNeil's I would give one-fourth grain and repeat the dose.

Dr. Underwood—I think we make a little mistake at times when we say that when the indicated remedy fails we should give something else. I think the indicated remedy in the proper potency will soothe the nerves quicker than anything else. A high potency of *Staphisagria* given before an operation will leave the patient in such a condition that he can hardly realize that an operation has been performed at all. I think the indicated remedy is the best pain-killer we have.

Dr. McNeil—The only thing is when we fail to give the indicated remedy. We may not be able to master the materia medica. I have a great deal of sympathy for the physician who finds himself in that position, and I do not blame him when he is in that fix if he gives a palliative, but I do not want him to get up at the next homœopathic meeting he attends and say that he could not cure with homœopathic remedies and had to use a palliative.

Dr. Augur—I agree with Dr. McNeil that the pain in the passage of a calculus is due to spasm of the ureter, and I give the Morphine to relieve the pain during this time.

Dr. McNeil—The Morphine is not necessary, for it has been proven that the indicated remedy has made the passage of calculi painless.

Dr. Augur—The fact that it did it in one case would not make it so in every case.

Dr. McNeil—We must admit that *we* fail often, but *Homœopathy never.*

Dr. Wilson—My experience with nephritic colic has not been enough to make me resort to Morphine. My indicated remedies have always relieved, not perhaps immediately or as soon as the Morphine would, but in time they have done so.

Dr. Augur—Any one who has ever suffered from this condition knows that at the end of say an hour your nerve gives out, and you must have something to relieve you.

Dr. McNeil—In what time in such a case of pain would you expect to relieve with Morphine?

Dr. Augur—Inside of twenty minutes.

Dr. McNeil—I can beat that with the indicated remedy. I have seen relief from the indicated remedy in five seconds. A patient, suffering with violent colic, told me that when the medicine was in her throat she had relief.

Dr. Augur—That is very true. In a case of mine of a young girl during the menstrual period suffering intensely with headache. She had consumptive symptoms and had been taking *Phosphorus,* which was her remedy, and I disliked to change it, as she was improving under it. I thought this remedy would relieve the headache also and I gave it to her in water, and the pain was relieved almost immediately. I simply cite this case to show that I have as much confidence in Homœopathy as any one.

Section 68 was read.

### Discussion.

Dr. McNeil—Here is one point that should have some attention. The poor patient or the vital-force has two enemies—namely, the morbid enemy and the palliative enemy.

Sections 69–71 was now read.

DISCUSSION.

Dr. Augur—Is it absolutely true that when you find a pathological condition, as abscess or ulcer of the stomach, that this is a dynamic disturbance of the vital-force?

Dr. McNeil—The abscess or ulcer is not the disease, but is the product of the disease.

Dr. Augur—I accept your explanation, but I do not think that Hahnemann makes it so clear.

The meeting was then declared adjourned, to meet again the first Friday in March at the office of Dr. J. M. Selfridge, in Oakland, when the reading of *The Organon* would be commenced at Section 70.

W. E. LEDYARD, *Secretary.*

Reported by Eleanor F. Martin, M. D.

